# Commencement in the Medical Institute

**Published:** 1846-04

**Authors:** 


### THE WESTERN JOURNAL.

New Series.—Vol. V.—No. IV.

LOUISVILLE, APRIL 1, 1846.

### COMMENCEMENT IN THE MEDICAL INSTITUTE.

At the eighth public Commencement in the Medical Insitute, held on the 9th of March last, the degree of Doctor of Medicine was conferred on seventy-three young gentlemen, who had undergone satisfactory examinations before the Faculty, and otherwise complied with the requirements of the institution. Of this number, twenty-one were from Kentucky, seventeen from Tennessee, eleven from Mississippi, and the remaining twenty-four from nearly half that number of States. Rarely, in our opinion, has a more gifted or promising body of young men gone forth from any of our medical schools than the last graduating class of the Institute. Many of them have given proofs of a strength and activity of mind, a ripeness of attainment, and a power of sustained application which betoken the happiest results. Every friend of sound medical learning will unite with us in the prayer, that these promises may be fulfilled, and that each succeeding class, in all our schools, may be an improvement upon all that have gone before.

The valedictory address to the graduates was delivered by Professor Miller, and was appropriate, judicious and eloquent. It inculcated in forcible language the code of ethics which must be observed in the professional intercourse of medical men, if they would have peace among themselves, or enjoy the respect of society. It urged with especial emphasis the duty of the physician to respect the professional character of his brethren, and never by word, look, or action attempt to disturb the confidence reposed in them by their patients.

In the conclusion of his address, Professor Miller adverted to the new title which the Medical Institute is about to assume in becoming a department of the University of Louisville. This, he stated, was the completion of the design originally entertained by the founders of the school. From the beginning, it was purposed to establish other departments in connexion with the Institute, and embrace all under a common charter. The charter which was granted by the Legislature at its last session was drawn up by the friends of the medical school, and fully secures to it all its emoluments, property, and privileges. Its friends have cause to congratulate themselves that it is thus placed beyond the reach of any further agitation, and may pursue an uninterrupted career of prosperity and usefulness.

The *honorary* degree of Doctor of Medicine was at the same time conferred on Mr. Josiah Gregg, of Missouri, author of '*Commerce of the Prairies;*' on Dr. A. W. Chapman, of Apalachicola, Florida; and on Dr. John S. Lewright, of Kentucky.

Robert M. Spencer, M. D., of Kentucky, a graduate of the Medical Department of Transylvania University, J. H. Crews, M. D., of Ohio, a graduate of the Medical College of Ohio, and Geo. A. J. Mayfield, M.D., of Tennessee, a graduate of the University of Pennsylvania, were admitted to the "*ad eundem*" Degree in the Medical Institute.

